# Effect of Ashwagandha (*Withania somnifera*) extract on sleep: A systematic review and meta-analysis

**DOI:** 10.1371/journal.pone.0257843

**Published:** 2021-09-24

**Authors:** Kae Ling Cheah, Mohd Noor Norhayati, Lili Husniati Yaacob, Razlina Abdul Rahman

**Affiliations:** Department of Family Medicine, School of Medical Sciences, Universiti Sains Malaysia, Kota Bharu, Kelantan, Malaysia; Institute of medical research and medicinal plant studies, CAMEROON

## Abstract

**Objective:**

To determine the effect of Ashwagandha extract on sleep.

**Methods:**

A comprehensive search was conducted in CENTRAL, MEDLINE, SCOPUS, Google Scholars, World Health Organization Trials Portal, ClinicalTrials.gov, Clinical Trial Registry of India, and AYUSH Research Portal for all appropriate trials. Randomized controlled trials that examined the effect of Ashwagandha extract versus placebo on sleep in human participants 18 years old and above were considered. Two authors independently read all trials and independently extracted all relevant data. The primary outcomes were sleep quantity and sleep quality. The secondary outcomes were mental alertness on rising, anxiety level, and quality of life.

**Results:**

A total of five randomized controlled trials containing 400 participants were analyzed. Ashwagandha extract exhibited a small but significant effect on overall sleep (Standardized Mean Difference -0.59; 95% Confidence Interval -0.75 to -0.42; I^2^ =  62%). The effects on sleep were more prominent in the subgroup of adults diagnosed with insomnia, treatment dosage ≥600 mg/day, and treatment duration ≥8 weeks. Ashwagandha extract was also found to improve mental alertness on rising and anxiety level, but no significant effect on quality of life. No serious side effects were reported.

**Conclusion:**

Ashwagandha extract appears to has a beneficial effect in improving sleep in adults. However, data on the serious adverse effects of Ashwagandha extract are limited, and more safety data would be needed to assess whether it would be safe for long-term use.

## Introduction

Sleep is a state of reversible unconsciousness in which the brain is less responsive to external stimuli [1]. Throughout the period of sleep, the body will cycle periodically between non-rapid eye movement (NREM) sleep and rapid-eye-movement (REM) sleep. NREM sleep is further divided into four stages, which is a continuum of relative depth [2]. Sleep episode in adults starts with a brief period of NREM stage 1, the lightest sleep stage. It progresses through NREM stage 2, stage 3 and stage 4, the deepest sleep stage. NREM sleep is then followed by REM sleep, which is known for the most vivid dreams and bodily movements. The cycle is repeated three to six times, and as sleep episode progresses, the duration of stages of NREM sleep shorten while the duration of REM sleep lengthens [3].

Healthy sleep is essential for neural development, learning, memory, cardiovascular and metabolic regulation. Sufficient sleep is needed to provide recovery after preceding waking activities and ensure optimal functioning during subsequent wakefulness [4]. As recommended by the National Sleep Foundation, in a healthy individual, the recommended sleep duration for younger adults is seven to nine hours, and for older adults is seven to eight hours [5]. In general, sleep duration decreases with increasing age. In a meta-analysis that involved 5273 healthy adults, total sleep time decreased by approximately 10 minutes for each decade of age [6]. However, there is no ideal duration of sleep required per night. A generally accepted assumption is that amount of sleep is enough if the individual wakes up feeling well-rested and perform well during the day [7].

Other than adequate duration, healthy sleep comprises good quality. A systematic review in 2016, involving 277 studies, has identified all possible sleep indicators and came out with the first sleep quality recommendation, which the National Sleep Foundation endorsed. Sleep continuity variables including 1) sleep latency of 15 minutes and less, 2) one or fewer awakening of more than five minutes per night, 3) wake time after sleep onset of 20 minutes and less, and 4) sleep efficiency of 85% and more are indicators of good sleep quality regardless of age [8]. However, no consensus reached regarding sleep architecture or nap-related variables as an indicator of good sleep quality. In general, studies have shown that the percentage of NREM sleep increases and the percentage of REM sleep decreases with increasing age. Older adults tend to have less consolidated sleep with more frequent awakenings [9].

Studies have reported a decline in sleep duration and sleep quality over the years in many modern societies. This is shown in a 27-year follow up study of United States adults where the percentage of adults sleeping six hours and less has increased by 31% from 1985 to 2012. In China, 11% of adults slept six hours and less per day [10–12]. While in an Italian study involving 3120 adults, 14% reported sleep dissatisfaction, and 30% has insufficient sleep [13]. Other than a decline in sleep quantity and quality, the prevalence of insomnia disorder also shows an increasing trend. It ranges from 3.9% to 22.1%, with an average of approximately 10% for multinational studies that used the Diagnostic and Statistical Manual of Mental Disorders 4^th^ edition (DSM-IV) criteria [14]. The incidence is even higher in older adults, where 50% reported having sleep problems [15].

Insomnia disorder is characterized by chronic dissatisfaction with sleep quantity or quality. It is defined in the Diagnostic and Statistical Manual of Mental Disorders 5th edition (DSM-5) as a subjective complaint of difficulty initiating sleep, difficulty maintaining sleep, or early morning awakenings that occur at a minimum of three nights per week for three months, and associated with one or more daytime symptom such as fatigue, cognitive impairment or mood disturbance. In comparison to the DSM-IV, the distinction into primary and secondary insomnia, whether the insomnia is comorbid with or caused by other disorders, was replaced by the term “insomnia disorder” in DSM-5. This shift reflects that insomnia is now recognized as an independent disorder [16].

Despite the progress made in understanding the aetiology and pathophysiology of the disorder, there is no universally accepted model. In 2015, Levenson and colleagues presented a model of the pathophysiology of insomnia after looking into findings at various levels of analysis [17]. They proposed that insomnia is most likely to develop in those who have a genetic vulnerability and have abnormalities in neurobiological processes. These vulnerabilities may lead to neurophysiologic hyperarousal and psychologic and behavioural processes, which, individually or together, increase an individual’s risk of developing insomnia and associated downstream health consequences. Precipitating stressors and other person-specific factors such as age and sex moderate these relationships. However, each of these processes varies among different individuals.

There is increasing evidence of the consequences of short sleep duration. Study shows that a short period of sleep restriction for three days can significantly increase sleepiness, fatigue, stress, and decreased functioning [18]. Several systematic reviews have reported the association between short sleep duration and the increased risk of hypertension [19, 20], type 2 diabetes mellitus [21], obesity [22], metabolic syndrome [23], coronary heart disease [24], and stroke [25]. A systematic review in 2017 has shown that short sleep duration, defined as less than six hours of sleep per 24 hours, is associated with a significant mortality increase [26]. All the studies demonstrated that an adequate amount and good-quality sleep are essential for overall quality of life (QoL).

Insomnia is generally treated using pharmacological and non-pharmacological approaches. Commonly used pharmacological agents include benzodiazepine receptor agonists, sedating antidepressants, sedating antihistamines, melatonin receptor agonists, and dual orexin receptor antagonists [27]. However, most of these available drugs might develop dependency and/or adverse effects. Hence, non-pharmacological treatment is generally considered the first-line treatment for a sleep disorder. Non-pharmacological treatments include cognitive-behavioural therapy for insomnia, sleep hygiene, psychotherapy, and relaxation techniques [28].

Although cognitive-behavioural therapy for insomnia is the preferred choice of non-pharmacological therapy for insomnia, it is underutilized because of a scarcity of its providers. Hence, complementary and alternative therapies gain increasing popularity in the treatment options of insomnia. These include massage, music therapy, aromatherapy, acupuncture and/or acupressure and herbal medicine. Sleep problems were rated one of the five most common conditions in people seeking complementary and alternative medicine treatments, including herbal products [29].

One of the herbal therapies currently under investigation for insomnia is *Withania somnifera*, commonly known as Ashwagandha. It has been used for centuries for various purposes in Ayurveda, the traditional system of medicine in India. It is an evergreen, straight, branching shrub that originates in Western India and Mediterranean regions [30]. The roots of plants are considered the most important part of the whole plant, as they are rich in bioactive molecule, especially withanolides, which is responsible for their medicinal property [31]. Other parts of the plant, such as its stem and leaves, also contain several bioactive molecules [32].

There is an increase in demand in Ashwagandha in the form of dietary supplements. Ashwagandha extracts from its root, leaves or combination of two is marketed in many forms, but pills and capsules are the commonest [30]. Researches have shown that it possesses anti-inflammatory, anti-cancer, anti-stress, anti-oxidant, immunomodulatory, hemopoietic and rejuvenating properties [33, 34]. Extracts obtained from the roots and leaves have been used for multiple purposes, such as rheumatic pain, joints inflammation, cognitive disorders, anxiety and stress disorders, male infertility, and improvement in physical performance [35–39].

Ashwagandha extract is generally well tolerated. A study has shown the haematological and biochemical organ function safety of Ashwagandha even in higher doses ranging from 6–10 gm of crude pulverized roots administered in aqueous extract form [40]. The exact mechanism is unknown. In an earlier study, Ashwagandha was thought to induce sleep through GABAergic activity, as seen in sleep-deprived rats [41]. Withanolides, the major biologically active constituents of Ashwagandha roots and leaves, are believed to be responsible for the majority of biological functions of Ashwagandha. However, Kaushik and colleagues noted ethanol extract that contains a high ratio of withanolides has failed to induce sleep in mice, indicating that withanolides might not be involved in sleep promotion [42]. A more recent study found that the triethylene glycol (TEG) in water extract is responsible for sleep in mice. TEG induces sleep by increasing the number of NREM episodes and decreasing the duration of the wake episode. However, studies on the effect of Ashwagandha related to insomnia in human are limited.

Poor sleep poses a great public health concern. It causes morbidities and huge economic loss to society through traffic accident, less productivity at work, and clinical cost for the treatment [43]. Although it is expected, sleep problem remained underdiagnosed and undertreated. Due to possible side effects from currently available pharmacological treatment for insomnia [27], herbals utilization for sleep problems has been growing. Although Ashwagandha extract has been reviewed extensively for its use in many medical purposes, its effect on sleep has not been reviewed. Hence, evaluation of the effects and safety of Ashwagandha on sleep can provide a basis for use decisions. The main objective of this review is to determine the effect of the Ashwagandha extract on sleep.

## Methods

The protocol was registered under the PROSPERO with the registration number CRD42021229064. This meta-analysis was conducted according to the PRISMA statement (Preferred reporting items for systematic reviews and meta-analyses) [44].

### Literature search

We systematically searched the following electronic databases until February 2021: 1) CENTRAL, 2) MEDLINE, 3) SCOPUS, 4) Google Scholars, 5) World Health Organization Trials Portal, 6) ClinicalTrials.gov., and 7) Clinical Trial Registry of India (CTRI). The keywords applied were (Ashwagandha OR Withania OR "Withania somnifera" OR "WS" OR "somniferas, Withania" OR "Indian ginseng" OR "winter cherry") AND (sleep OR insomnia OR "difficulty sleeping" OR "disorder of sleep initiation and maintenance" OR "sleep initiation and maintenance disorder" OR "DIMS" OR "sleep wake disorder" OR "SWD" OR "sleep quality" OR "quality of sleep" OR "sleep index" OR "sleep scale" OR "sleep time" OR "actigraphy").

Searches in Google and in the AYUSH Research Portal were performed to assess the extent of grey or unpublished literature on the topic. Pharmaceutical companies including Ixoreal Biomed and NutriScience that market Ashwagandha were contacted to identify unpublished trials. In addition, the reference lists of all eligible articles and related reviews were also hand-searched to avoid any missing studies. The process above was performed repeatedly to include additional eligible studies. Only studies published in the English language were included, but no restriction was conducted based on publication date.

### Study eligibility

Studies were included if they met these inclusion criterias: 1) human randomized controlled trials, 2) study participants over 18 years old with or without insomnia, 3) intervention using Ashwagandha extract in any form, any dose, or any duration, and 4) evaluated at least one outcome on sleep. Those with insomnia should be diagnosed by standard diagnostic criteria such as the Diagnostic and Statistical Manual of Mental Disorders (DSM) or the International Statistical Classification of Diseases and Related Health Problems (ICD). The primary outcome of this review was to determine the effect of the Ashwagandha extract on sleep, referring to sleep quantity and quality. These may consist of self-rating scale on sleep, sleep quality index, sleep efficacy score, symptom assessment scale, actigraphy sleep parameters, polysomnography sleep parameters or sleep log. The secondary outcomes included in this review were mental alertness on rising, anxiety level, QoL, and safety of the intervention, which was assessed as the number of participants with adverse events.

### Data extraction

Two authors (CKL and RAR) independently screened titles and abstracts and assessed the full text of potentially eligible studies. Studies were selected if they met the inclusion criteria. At each stage of screening and assessing the eligibility of studies, we excluded trials which were: 1) non-clinical trials, 2) animal studies, and 3) examining the effect of Ashwagandha extract along with other interventions. Review papers, abstracts in conferences, book chapters, and duplicate studies were also excluded.

The data of interest were extracted by two authors (CKL and RAR). The collected data from each eligible study included the following information: 1) study details (first author, year of publication, country), 2) study design, 3) sample size, 4) patient characteristics (mean age, health status), 5) intervention group details (form, dosage), 6) control group details, 7) duration of the study, and 8) outcome measures. Any discrepancy was resolved by discussion with the third author (LHY). Wherever study reports allowed, we used the data from the intention-to-treat analysis. We contacted Deshpande et al to request missing information from their study [45].

### Risk of bias

We assessed the risk of bias based on random sequence generation, allocation concealment, blinding of participants and personnel, blinding of outcome assessors, completeness of outcome data, selectivity of outcome reporting, and other bias, as discussed in the Cochrane Handbook for Systematic Reviews of Interventions [46]. We resolved any disagreements by discussion.

### Statistical analysis

Forest plots were drawn for the trials with continuous outcomes using mean differences (MD) and 95% confidence intervals (CI). For trials using different measurement tools, standardized mean difference (SMD) was calculated as the difference in means between groups divided by the pooled standard deviation. For dichotomous outcomes, we analyzed using risk ratios (RR) and 95% CI. Included trials were checked for unit of analysis errors and we did not encounter any of these. If we had encountered any cluster-RCTs, we intended to adjust the results from trials showing unit of analysis errors based on the mean cluster size and intra-cluster correlation coefficient [46]. We contacted the original trial authors to request missing or inadequately reported data. We performed analyses based on the available data in the event that missing data were not available. Heterogeneity was assessed in two steps. First, we assessed obvious heterogeneity at face value by comparing populations, settings, interventions and outcomes. Second, we assessed statistical heterogeneity by means of the I^2^ statistic [46]. If there were sufficient studies, we intended to use funnel plots to assess the possibility of reporting biases or small study biases, or both.

Meta-analyses were performed using Review Manager 5.4 software (RevMan 2020). We used a random-effects model to pool data. Thresholds for the interpretation of the I^2^ statistic can be misleading, since the importance of inconsistency depends on several factors. We used the guide to interpret heterogeneity as outlined in the Cochrane Handbook for Systematic Reviews of Interventions: 0% to 40% might not be important, 30% to 60% may represent moderate heterogeneity, 50% to 90% may represent substantial heterogeneity, and 75% to 100% would be considerable heterogeneity [46]. The planned subgroup analyses were: 1) sleep by type of measurements, 2) participant’s age 18–59 years old or 60 years old and above, 3) participant’s underlying background with insomnia or without insomnia, 4) form of Ashwagandha extract in pill or capsule or liquid, 5) dose of Ashwagandha extract, and 6) duration of Ashwagandha extract administration. However, we were unable to carry out all subgroup analyses in the categories outlined in the protocol as there were insufficient data. We only managed to perform subgroup analyses on: 1) sleep by type of measurements, 2) participant’s underlying background with insomnia or without insomnia, 3) dose of Ashwagandha extract, and 4) duration of Ashwagandha extract administration.

Sensitivity analysis was performed to investigate the impact of risk of bias for sequence generation and allocation concealment of included studies. We also assessed the quality of evidence for primary and secondary outcomes according to Grades of Recommendation, Assessment, Development and Evaluation (GRADE) methodology for risk of bias, inconsistency, indirectness, imprecision, and publication bias [47]. The GRADE approach specifies four levels of quality, the highest of which is for randomized trial evidence. It can be downgraded to moderate, low or even very low-quality evidence.

## Results

### Results of the search

**Fig 1:**
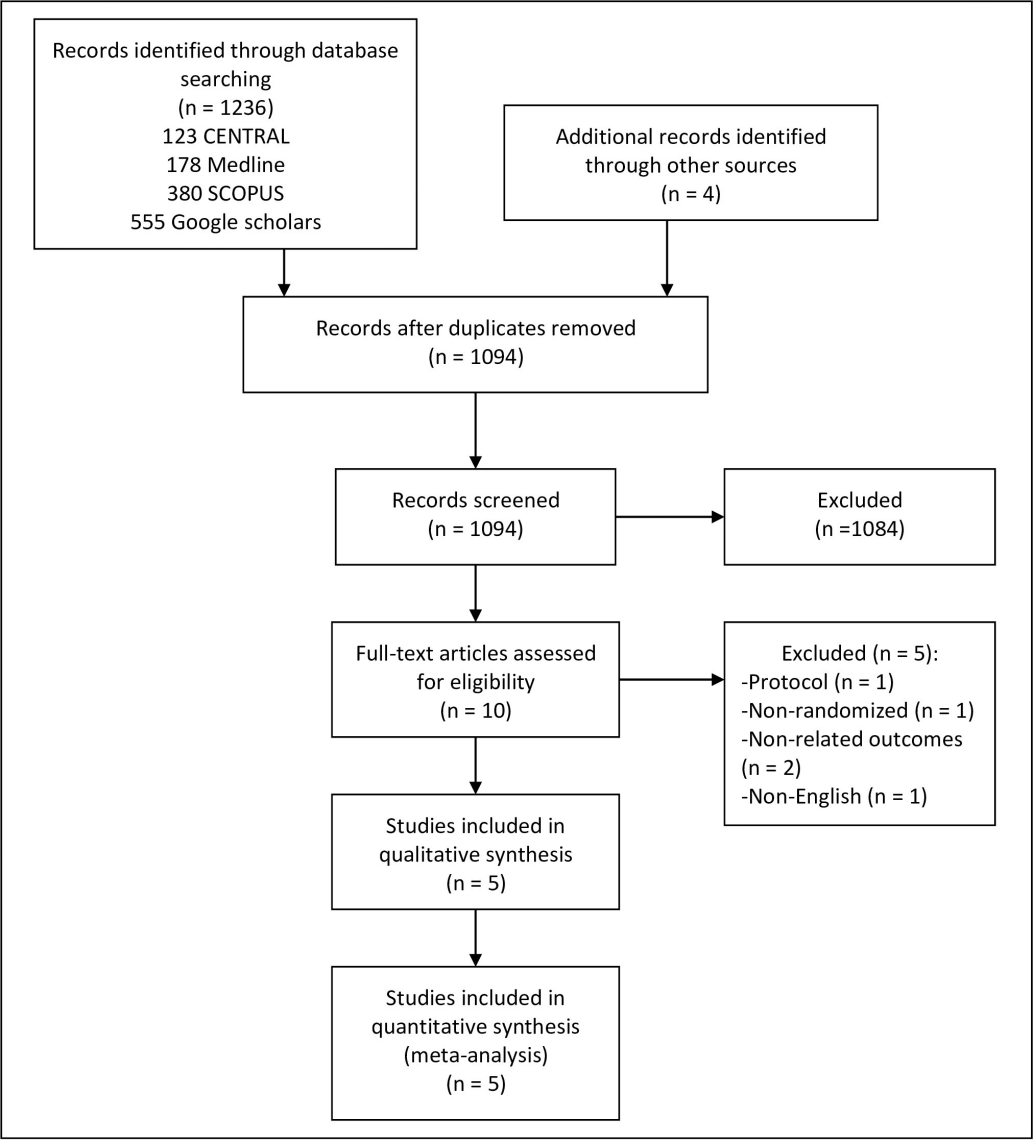
Study flow diagram.

A total of 1236 records were returned from the comprehensive database search. In addition, four records were identified through sources outside of the core search: one through reference list searching and three through AYUSH Research Portal. Upon elimination of duplicates, a total of 1094 records were screened for eligibility. Fig 1 further depicts the selection process of the studies. After completion of screening, 10 full-text articles were assessed for eligibility. Five trials that met the pre-specified inclusion criteria were selected for this systematic review and meta-analysis. We had to exclude five trials from this review due to: one study protocol [48], one trial non-randomized [49], two trials with outcomes not related to sleep [50, 51], and one trial due to non-English language [52].

### Characteristics of included studies

We included five trials with a total of 400 participants [45, 53–56]. Table 1 further describes the studies included. Out of the five trials, one trial declared that their study was partially sponsored by the manufacturer [45]. All trials were conducted in India [45, 53–56]. Three of the trials recruited participants from outpatient clinics [54–56], one trial recruited through phone call [45], and one trial did not mention the method of recruitment [53]. The five trials included in this meta-analysis comprised of two trials with 210 healthy adults [45, 53], one trial with 50 healthy elderly over 60 years old [55], one trial with 60 adults diagnosed with insomnia based on DSM-IV [54], and one trial that included two groups of participants which were 40 healthy adults, and 40 adults diagnosed with insomnia based on DSM-IV [56]. Both sexes were included in all the trials. There was no baseline imbalance in demographic characteristics across the intervention and comparison groups in all five trials [45, 53–56].

**Table 1:** Characteristic of included studies.

Author (Publish year)	Study design	Sample size (Drop-outs)	Patient’s characteristics		WS Form/ Route/ Daily dose	Control	Duration (follow up interval)	Outcomes of interest		Conclusion of effect WS on sleep
			Health status	Mean age (SD)				Sleep outcomes	Other outcomes	
Salve et al (2019)	Randomize, DB, PC	I_1_: 19 (1) I_2_: 20 (0) C:19 (1)	Stressed healthy adults (PSS ≥20)	I_1_: 29.65 (6.36)	I_1_: Root extract in capsule (KSM-66)/ Oral/ 250 mg	Placebo capsule (starch power)	8 weeks (week 4, 8)	SQS	HAMA-A	Beneficial
				I_2_: 32.70 (8.79)						
				C: 30.35 (6.50)						
					I_2_: Root extract in capsule (KSM-66)/ Oral/ 600 mg					
Langade et al (2019)	Randomize, DB, PC	I: 39 (1)	Insomnia (DSM-IV)	I: 38.83 (5.00)	Root extract in capsule (KSM-66)/ Oral/ 600 mg	Placebo capsule (starch power)	10 weeks (week 5, 10)	SQS, PSQI, SOL, TST, WASO, TIB, Sleep efficiency	Mental alertness scale, HAM-A	Beneficial
		C: 19 (1)		C: 40.00 (6.21)						
Kelgane et al (2020)	Randomize, DB, PC	I: 19 (6)	Healthy elderly adults	I: 72.16 (4.36)	Root extract in capsule (KSM-66)/ Oral/ 600 mg	Placebo capsule (starch power)	12 weeks (week 4, 8, 12)	SQS	Mental alertness scale, QoL	Beneficial
		C: 20 (5)		C: 70.70 (4.47)						
Deshpande et al (2020)	Randomize, DB, PC	I: 71 (4)	Healthy adults	I: 36.8 (10.98)	Root and leave extract in capsule (Shoden®)/ Oral/ 120 mg	Placebo capsule (rice powder)	6 weeks (week 6)	SOL, TST, WASO, TIB, Sleep efficiency	QoL	Beneficial
		C: 73 (2)		C: 37.61 (10.32)						
Langade et al (2021)	Randomize, DB, PC	I: 18 (2)	P_1_: Healthy	I: 35.60 (8.74)	Root extract in capsule (KSM-66)/ Oral/ 600 mg	Placebo capsule (starch power)	8 weeks (week 1, 4, 8)	SQS, PSQI, SOL, TST, WASO, TIB, Sleep efficiency	Mental alertness scale, HAM-A	Beneficial
		C: 18 (2)	adults	C: 38.25 (7.83)						
		I: 20 (0)	P_2_: Insomnia	I: 38.70 (7.15)						
		C: 17 (3)	(DSM-IV)	C: 35.95 (5.49)						

C- Control group, DB- Double-blind, DSM- Diagnostic and Statistical Manual, HAMA-A- Hamilton-Anxiety scale, I- Intervention, PC- Placebo control, PSQI- Pittsburgh sleep quality index, P- population, QoL- Quality of life, SD- Standard deviation, SOL- Sleep Onset Latency, SQS- Sleep Quality Scale, TIB- Total Time In Bed, TST- Total Sleep Time, WASO- Wake Time After Sleep Onset, WS- *Withania somnifera*

Participants in the trials were randomized into intervention and control groups. For all five trials, the intervention was Ashwagandha extract in capsule form. Four trials with 250 participants used KSM-66 which contains extract from the root of the plant [53–56], and one trial with 150 participants used Shoden® which contains extract from both the root and leaves of the plant [45]. All trials instructed their participants to consume Ashwagandha extract capsule, either with milk or water, throughout their study duration. One trial used Ashwagandha extract in the dose of 120 mg per day [45], one trial compared Ashwagandha extract in two doses 250 mg per day and 600 mg per day with the control group [53], and three trials used Ashwagandha extract in the dose of 600 mg per day [54–56]. Out of the five trials, one trial was conducted for six weeks [45], two for eight weeks [53, 56], one for 10 weeks [54], and one for 12 weeks [55]. All the five trials that were included in this meta-analysis used placebo in the capsule form. Four used starch powder [53–56], and one used rice powder as a placebo in their study [45]. Placebo capsules were described as similar to intervention capsules in the context of shape, size, and colour in all four trials [53–56] but was not described in details in one of the trials [45].

Sleep was the primary outcome of our review. We have determined that the Sleep Quality Scale using seven-point scale and Pittsburgh Sleep Quality Index are subjective assessments; actigraphy parameters: sleep onset latency, total sleep time, wake time after sleep onset, total time in bed, and sleep efficiency as objective assessments of sleep. The Sleep Quality Scale using seven-point scale was reported by four trials [53–56], Pittsburgh Sleep Quality Index was reported by two trials [54, 56], and actigraphy parameters were reported by three trials [45, 54, 56]. Two of the trials reported a Sleep Quality Scale, Pittsburgh Sleep Quality Index, together with actigraphy parameters [54, 56].

The Sleep Quality Scale is a seven-point scale as perceived by the patient after waking up in the morning. It ranges from 1–7; the lower the score, the better perceived the sleep quality. Pittsburgh Sleep Quality Index is a self-administered questionnaire composed of seven components: subjective sleep quality, sleep latency, sleep duration, habitual sleep efficiency, sleep disturbances, use of sleep-promoting medications, and daytime dysfunction over one month. The summed up global score ranges from 0–21; the lower the score, the better the sleep quality. On the other hand, actigraphs are devices generally placed on the wrist to record movement. The collected data are then downloaded for analysis of activity/inactivity, and in turn, further generating sleep parameters such as sleep onset latency, total sleep time, wake time after sleep onset, total time in bed, and sleep efficiency. Sleep onset latency is the number of minutes between lying down in bed and actually falling asleep; total sleep time is the duration from sleep onset to sleep offset; wake time after sleep onset refers to the number of minutes a participant was awake between sleep onset and sleep offset; total time in bed is the duration a participant spent in bed and is derived by subtracting the time the subject went to bed from the time the subject arose, and sleep efficiency is defined as the ratio of total sleep time to total time in bed. Sleep onset latency of 15 minutes and less, wake time after sleep onset of 20 minutes and less, and sleep efficiency of 85% and above are indicators of good sleep quality [8]. Optimal sleep quantity has been shown to differ across the lifespan [5].

The secondary outcomes included in our review were mental alertness on rising, which was reported by three trials [54–56], anxiety level by Hamilton Anxiety Rating Scale (HAM-A) by three trials [53, 54, 56], and QoL by World Health Organization Quality of Life (WHOQOL-BREF) self-administered questionnaire by two trials [45, 55]. Mental alertness using a three-point scale is a valid tool to assess alertness as perceived by the patient after waking up in the morning. The scale ranges from 1–3; the lower the score, the better perceived mental alertness upon waking up in the morning. HAMA-A is a reliable tool to assess the intensity of anxiety, ranges from 0–56; the lower the score, the lower the intensity of anxiety. WHOQOL-BREF is a self-administered questionnaire with four domains. Scores obtained in each domain are scaled in a positive direction. The higher the score, the better QoL. All trials reported safety-related events and no serious adverse effects were reported in all five trials. One trial reported mild adverse events: one viral fever, one headache, two acid reflux, two allergic dermatitis in the Ashwagandha group [45].

Out of the five trials, one trial assessed the effect immediately after the intervention at week six [45], other trials with follow-ups of one [56], four [53, 55, 56], five [54], eight [53, 55, 56], 10 [54], and 12 weeks [55].

### Risk of bias in included studies

**Fig 2:**
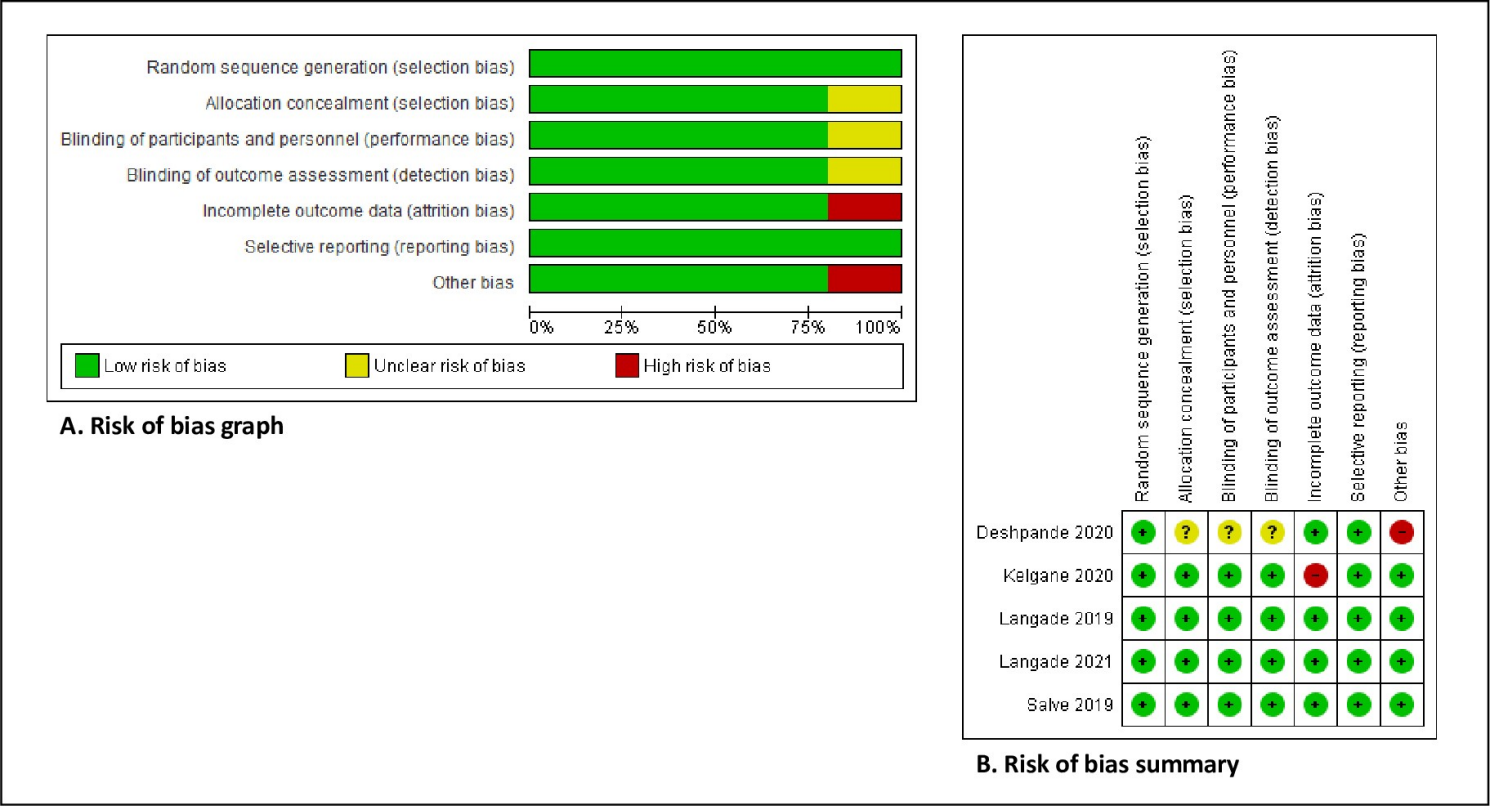
(A) Risk of bias graph: Review authors’judgements about each risk of bias item presented as percentages across all included studies, (B) Risk of bias summary: Review authors’ judgements about each risk of bias item for each included study.

The assessment of risk of bias is shown in Fig 2. Fig 2(A) shows the proportion of studies assessed as low, high or unclear risk of bias for each risk of bias indicator. Fig 2(B) shows the risk of bias indicators for individual studies. The method of randomization was described by all five trials. All of them used computer-generated randomization [45, 53–56]. Allocation concealment methods were described in four of the trials [53–56]. However, concealment of the allocation was not mentioned in one trial [45], and thus, we judged allocation concealment an unclear risk of bias. All the five trials included in this review used a placebo control. Blinding of participants and personnel and blinding of outcome assessment were described in four of the trials [53–56]. In one of the trials [45], blinding of personnel and blinding of outcome assessment were not described, and thus, we judged blinding an unclear risk of bias.

Primary outcomes were reported in all five trials. Two trials carried out the intention-to-treat analysis, in which the participants were analyzed according to the group that they were initially assigned [45, 53]. The other three trials carried out per-protocol analysis [54–56]. We judged a high risk of bias for incomplete outcome data for one trial [53], as the per-protocol analysis was performed, and the missing data was due to non-compliance to intervention, even though the missing data was less than 30% and it was balanced between the groups. Low risk of bias was judged for the others as the loss to follow-up in the intervention group was less than 20% [54, 56]. All trials reported the outcomes as specified in their methods section. Only two trials were prospectively registered in the Clinical Trials Registry-India www.ctri.nic.in [45, 56]. However, we found that in one trial, the dose of Ashwagandha used in the actual study was much lower than indicated in the protocol [45].

### Primary outcomes

Sleep measurements included in our analysis were: Sleep Quality Scale, Pittsburgh Sleep Quality Index, sleep onset latency, total sleep time, wake time after sleep onset, total time in bed, and sleep efficiency. In view of the difference in the trial duration and follow-up period, our meta-analysis aimed to evaluate the immediate post-treatment effects of Ashwagandha extract on sleep. Outcome measures that were conducted by two or more trials were analyzed in this review. Random effects model was applied to analyze different outcomes. As different measurements were used to measure the outcome on sleep, SMD was used. However, due to the difference in the direction of effect, -1 was multiplied for SMD of three measurements: total sleep time, total time in bed, and sleep efficiency. Hence, lower scores indicated better sleep. Our meta-analysis of pooled data showed that Ashwagandha extract has significant improvement in overall sleep compared to placebo (SMD -0.59; 95% CI -0.75 to -0.42; I^2^ =  62%; p <0.001; five trials; 1764 participants; moderate quality evidence) (Table 2).

**Table 2:** Summary of findings by GRADE quality assessment.

Outcomes	No of studi-es	Certainty assessment						No of patients		Absolute effect (95% Cl)	Consistency
		Study design	Risk of bias	Inconsis- tency	Indirect- ness	Impreci- sion	Other consider- ations	WS	Placebo		
Overall sleep	5	Random-ized trials	Not serious	Serious^a^	Not serious	Not serious	None	972	792	SMD **0.59 lower** (0.75 lower to 0.42 lower)	⨁⨁⨁◯ MODERATE
Sleep by Sleep Quality Scale	4	Random-ized trials	Not serious	Serious^a^	Not serious	Serious^b^	None	135	93	SMD **1.16 lower** (1.65 lower to 0.66 lower)	⨁⨁◯◯ LOW
Sleep by sleep onset latency	3	Random-ized trials	Not serious	Not serious	Not serious	Serious^b^	None	152	129	SMD **0.53 lower** (0.77 lower to 0.29 lower)	⨁⨁⨁◯ MODERATE
Sleep by total sleep time	3	Random-ized trials	Not serious	Not serious	Not serious	Serious^b^	None	152	129	SMD **0.45 lower** (0.69 lower to 0.21 lower)	⨁⨁⨁◯ MODERATE
Sleep by wake time after sleep onset	3	Random-ized trials	Not serious	Not serious	Not serious	Serious^b^	None	152	129	SMD **0.39 lower** (0.62 lower to 0.15 lower)	⨁⨁⨁◯ MODERATE
Sleep by sleep efficiency	3	Random-ized trials	Not serious	Serious^a^	Not serious	Serious^b^	None	152	129	SMD **0.68 lower** (1.07 lower to 0.29 lower)	⨁⨁◯◯ LOW
Sleep by treatment duration- ≥8 weeks	5	Random-ized trials	Not serious	Not serious	Not serious	Not serious	None	972	792	SMD **0.37 lower** (0.46 lower to 0.27 lower)	⨁⨁⨁◯ MODERATE

CI- Confidence interval, MD- Mean difference, SMD- Standardized mean difference, WS- *Withania somnifera*

Explanations: a. Substantial heterogeneity noted; b. Small sample size

### Evaluation of subgroup analysis

#### Sleep by type of measurements

**Fig 3:**
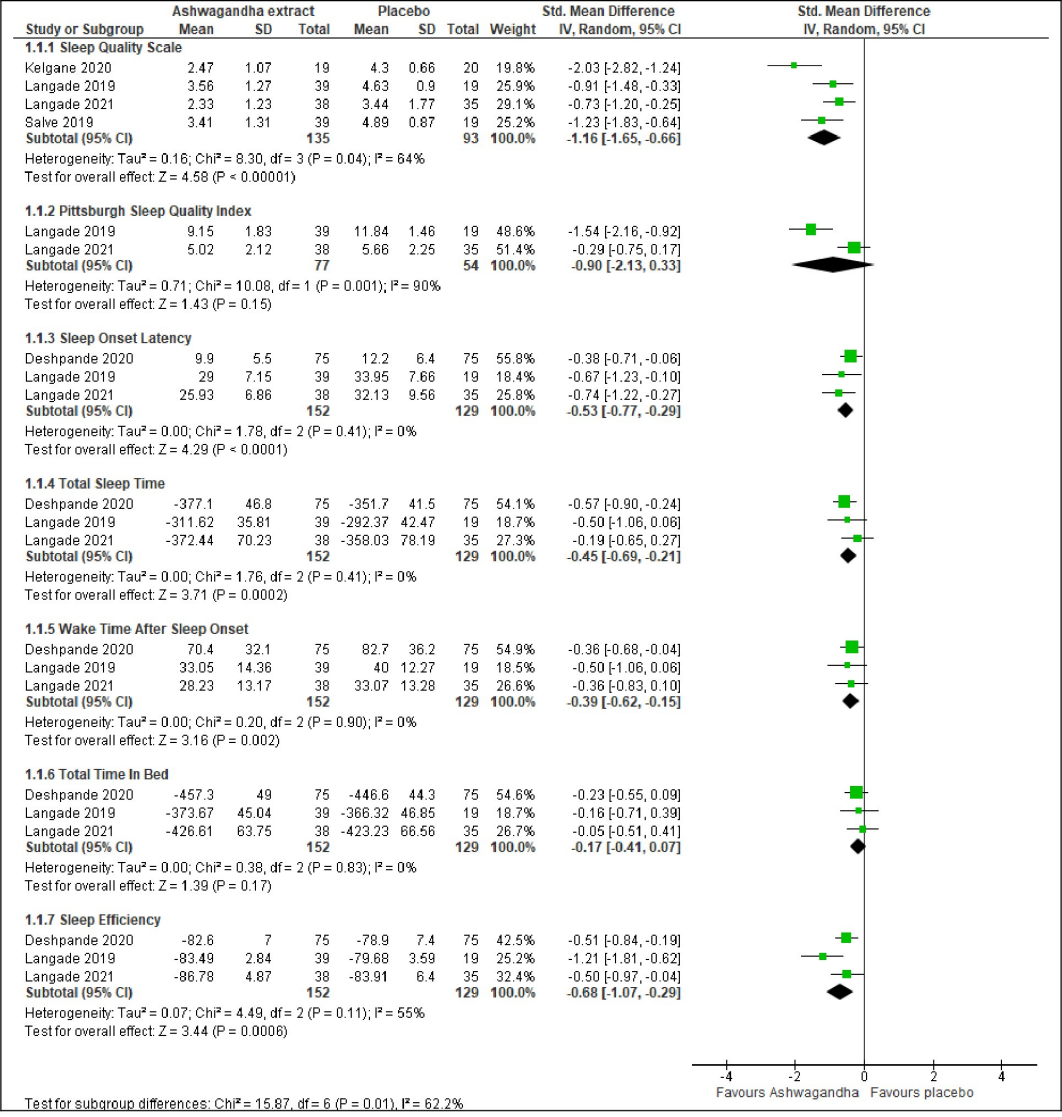
Forest plots of Ashwagandha versus placebo on sleep by type of measurements.

Significant evidence where Ashwagandha extract showed improvement in sleep compared to the placebo was noted for: Sleep Quality Scale using seven-point scale (SMD -1.16; 95% CI -1.65 to -0.66; I^2^ = 64%; p <0.001; four trials; 228 participants; low quality evidenve), sleep onset latency (SMD -0.53; 95% CI -0.77 to -0.29; I^2^ = 0%; p <0.001; three trials; 281 participants; moderate quality evidence), total sleep time (SMD -0.45; 95% CI -0.69 to -0.21; I^2^ = 0%; p <0.001; three trials; 281 participants; moderate quality evidence), wake time after sleep onset (SMD -0.39; 95% CI -0.62 to -0.15; I^2^ = 0%; p = 0.002; three trials; 281 participants; moderate quality evidence), and sleep efficiency (SMD -0.68; 95% CI -1.07 to -0.29; I^2^ = 55%; p <0.001; three trials; 281 participants; low quality evidence) (Fig 3, Table 2). However, there was no significant effect of Ashwagandha extract compared to placebo in improving Pittsburgh Sleep Quality Index (SMD -0.90; 95% CI -2.13 to 0.33; I^2^ = 90%; p = 0.150; two trials; 131 participants), and total time in bed (SMD -0.17; 95% CI -0.41 to 0.07; I^2^ = 0%; p = 0.170; three trials; 281 participants) (Fig 3).

#### Sleep by participant’s background with or without insomnia

**Fig 4:**
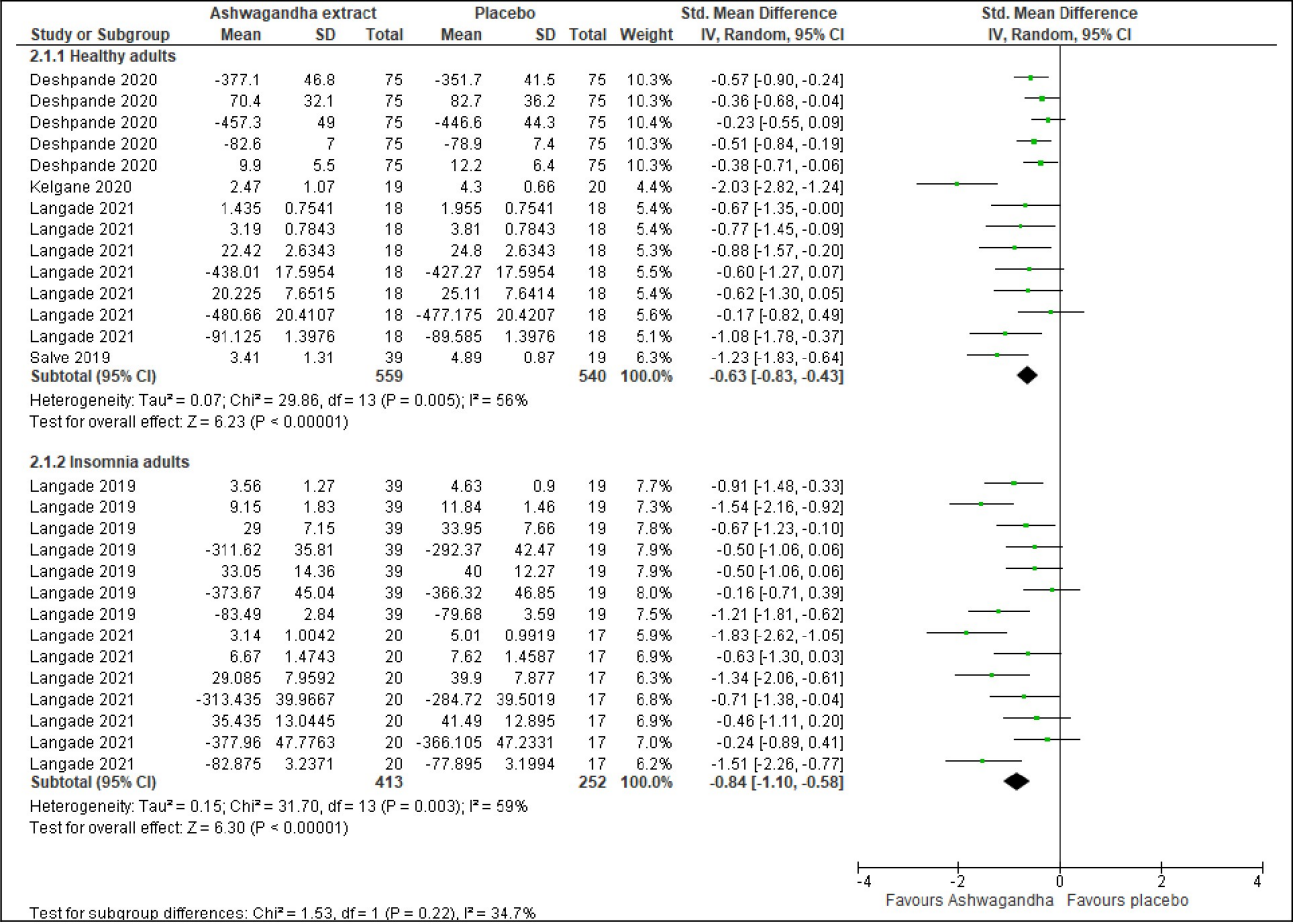
Forest plots of Ashwagandha versus placebo on sleep by participant’s background with or without insomnia.

Four trials involving 1099 healthy adults [45, 53, 55, 56] showed Ashwagandha extract has significant improvement in overall sleep compared to placebo (SMD -0.63; 95% CI -0.83 to -0.43; I^2^ =  56%; p <0.001) (Fig 4). On the other hand, two trials involving 665 adults diagnosed with insomnia [54, 56] also revealed a significant improvement in overall sleep in the Ashwagandha extract compared to the placebo group (SMD -0.84; 95% CI -1.10 to -0.58; I^2^ =  59%; p <0.001) (Fig 4).

#### Sleep by treatment dosage

**Fig 5:**
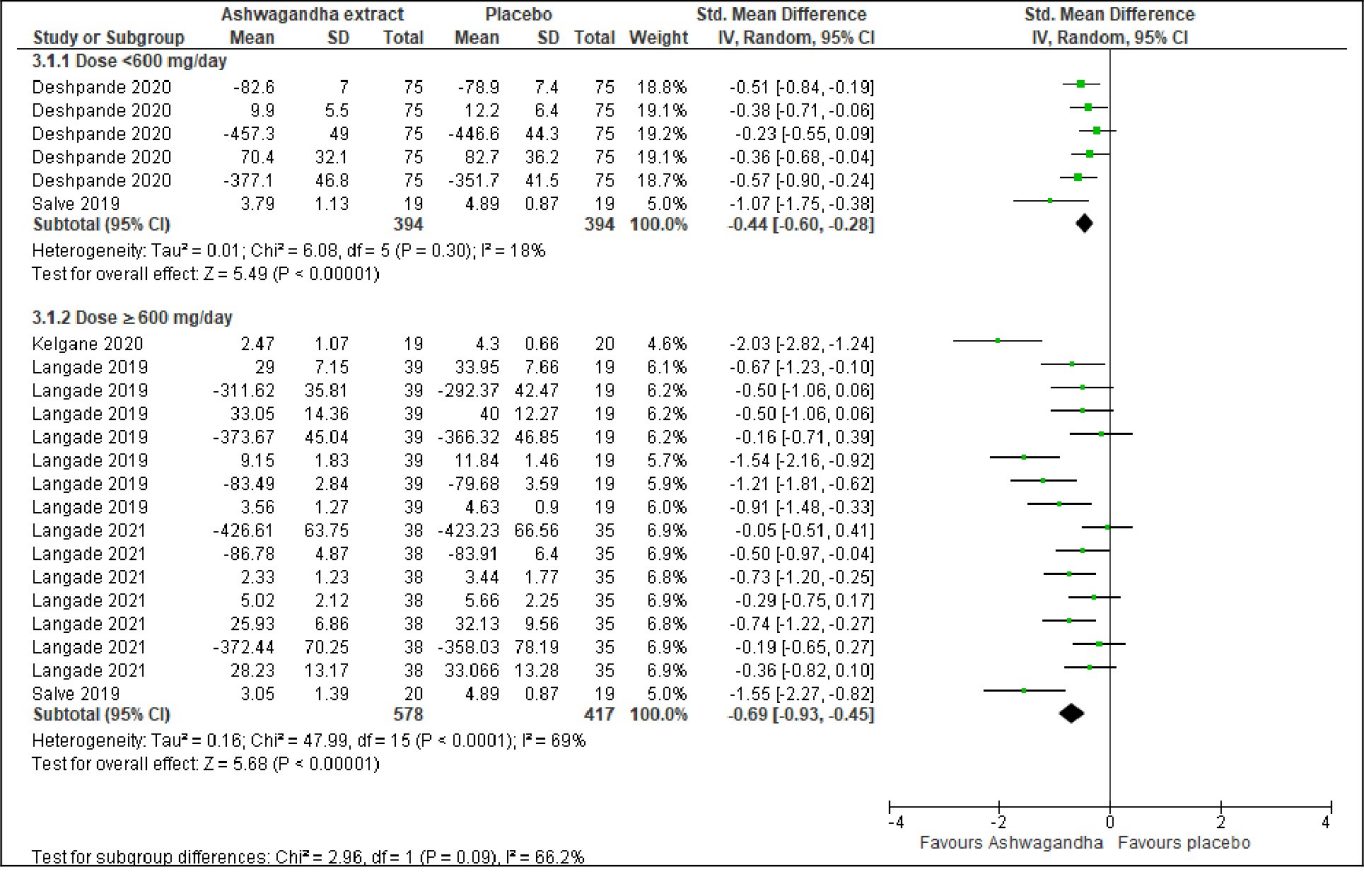
Forest plots of Ashwagandha versus placebo on sleep by treatment dosage.

Two trials involving 788 participants [45, 53] that studied Ashwagandha extract in the dosage of <600 mg/day revealed that Ashwagandha extract lead to a significant improvement in overall sleep compared to placebo (SMD -0.44; 95% CI -0.60 to -0.28; I^2^ =  18%; p <0.001) (Fig 5). Apart from that, four trials involving 995 participants [53–56], which studied Ashwagandha extract in the dosage of ≥600 mg/day, also showed Ashwagandha extract lead to a significant improvement in overall sleep compared to placebo (SMD -0.69; 95% CI -0.93 to -0.45; I^2^ =  69%; p <0.001) (Fig 5).

#### Sleep by treatment duration

**Fig 6:**
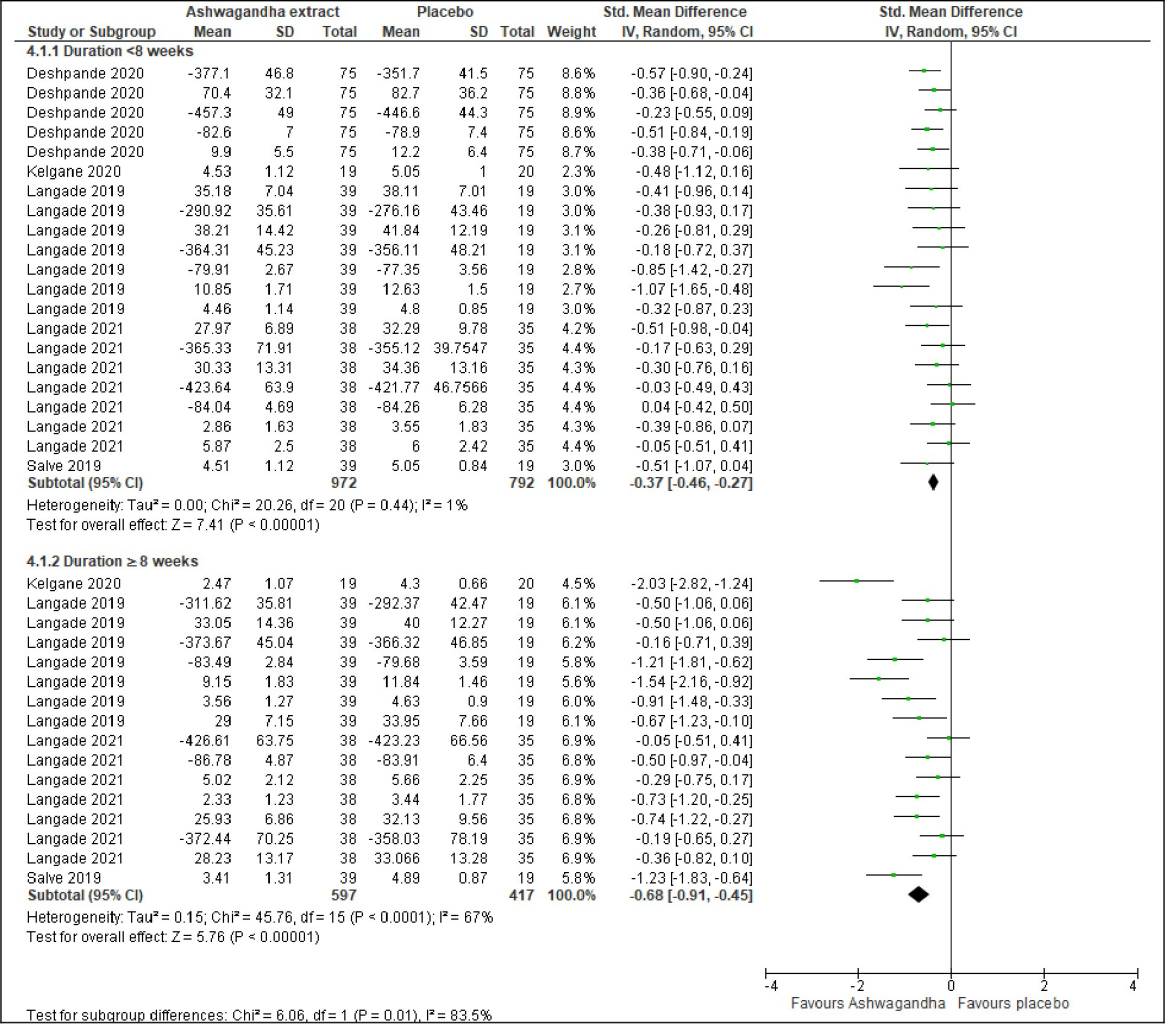
Forest plots of Ashwagandha versus placebo on sleep by treatment duration.

Five trials involving 1764 participants [45, 53–56] reported a significant improvement in overall sleep in the Ashwagandha extract compared to the placebo group with a treatment duration of <8 weeks (SMD -0.37; 95% CI -0.46 to -0.27; I^2^ =  1%; p <0.001) (Fig 6). Besides, four trials involving 1014 participants [53–56] also revealed a significant improvement in overall sleep in the Ashwagandha extract compared to the placebo group with a treatment duration of ≥8 weeks (SMD -0.68; 95% CI -0.91 to -0.45; I^2^ =  67%; p <0.001; moderate quality evidence) (Fig 6, Table 2).

### Secondary outcomes

**Fig 7:**
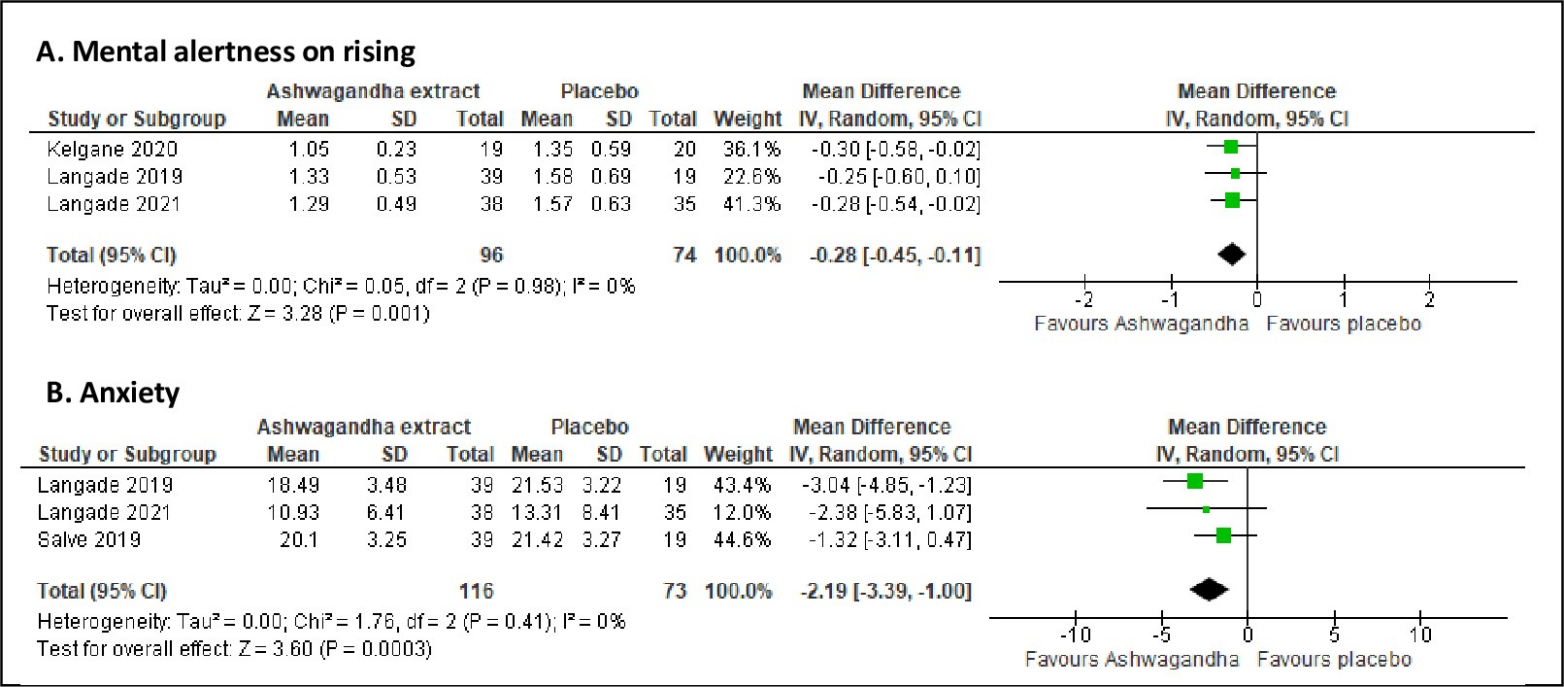
Forest plots of Ashwagandha versus placebo on (A) mental alertness on rising, (B) anxiety.

**Fig 8:**
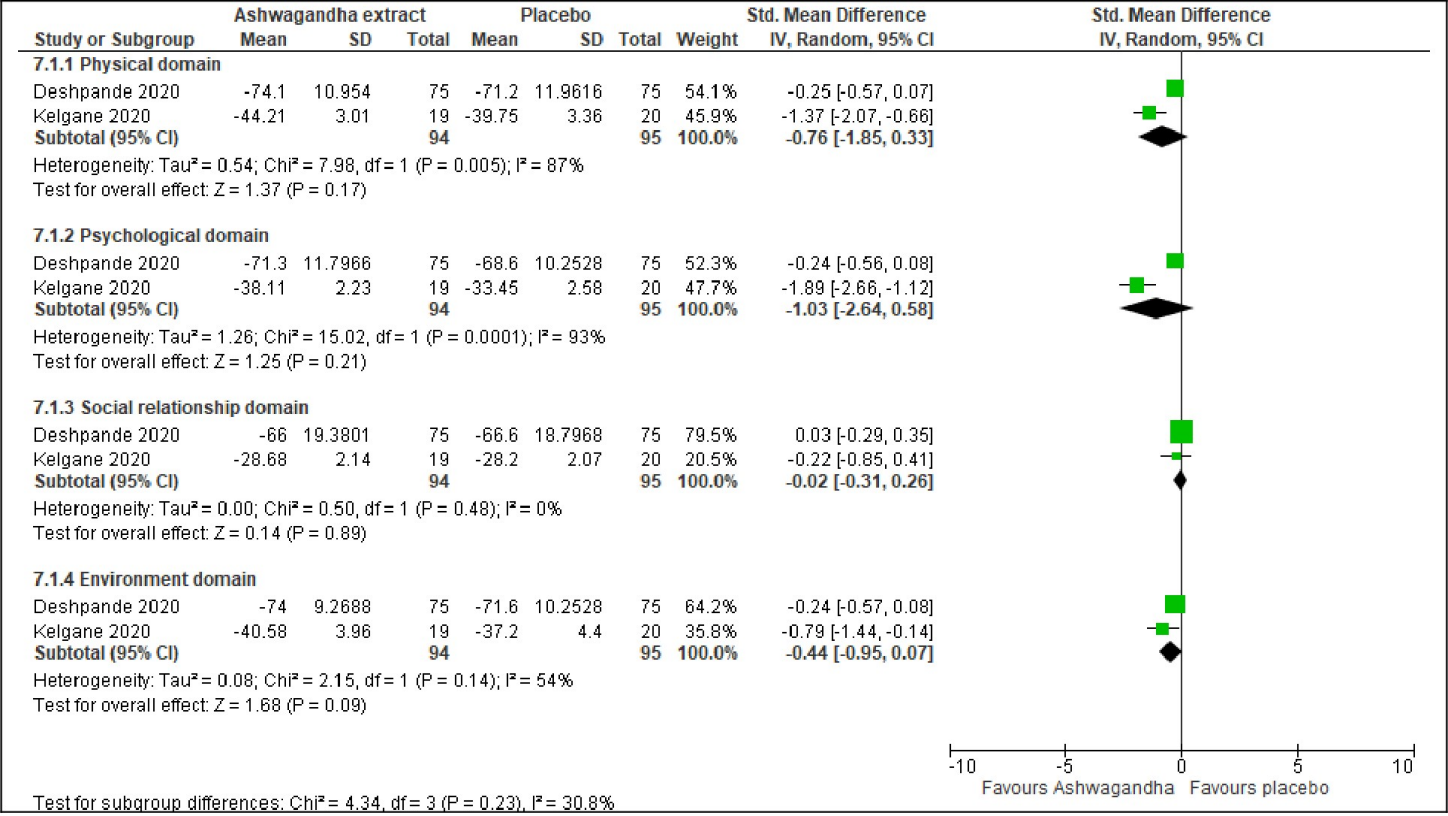
Forest plots of Ashwagandha versus placebo on quality of life.

Mental alertness on rising was reported by three trials, involving 170 participants [54–56]. The meta-analysis showed a significant improvement in mental alertness compared to placebo (MD -0.28; 95% CI -0.45 to -0.11; I^2^ = 0%; p = 0.001). (Fig 7(A)). Anxiety level was reported by three trials, involving 189 participants [53, 54, 56]. The meta-analyses showed significant improvement in anxiety level (MD -2.19; 95% CI -3.39 to -1.00; I^2^ = 0%; p <0.001) (Fig 7(B)). On the other hand, QoL was reported by two trials that involved 189 participants [45, 55]. The meta-analysis revealed no significant improvement in QoL in term of four domains: physical domain (SMD -0.76; 95% CI -1.85 to 0.33; I^2^ = 87%; p = 0.170), psychological domain (SMD -1.03; 95% CI -2.64 to 0.58; I^2^ = 93%; p = 0.210), social relationship domain (SMD -0.02; 95% CI -0.31 to 0.26; I^2^ = 0%; p = 0.890), and environment domain (SMD -0.44; 95% CI -0.95 to 0.07; I^2^ = 54%; p = 0.090) (Fig 8).

### Sensitivity analysis

There was no substantial change in the effect sizes and confidence interval for trials with unclear risk of bias for allocation concealment and random sequence generation for all outcomes.

### Differences between protocol and review

We included a subgroup analysis for the type of sleep measurements as we think there might be a difference in the different measurement. We changed the intended subgroup of age group analysis from 65 years and above to 60 years and above, as the only trial that studied on elderly population recruited patients 60 years old and above [55]. However, we cannot proceed with subgroup analysis for different age group due to insufficient trials.

## Discussion

This review was designed to examine the evidence for the effect and safety of Ashwagandha extract on sleep in adults. The meta-analysis of five trials revealed evidence for a clinically beneficial effect of Ashwagandha extract compared to placebo in improving sleep. A small but significant improvement in overall sleep was observed with a reduction of 0.59 units. The evidence of effect was noted for Sleep Quality Scale using seven-point scale, sleep onset latency, total sleep time, wake time after sleep onset, and sleep efficiency. The effects were more prominent in adults diagnosed with insomnia, treatment dose ≥600 mg/day, and treatment duration ≥8 weeks. However, there were insufficient trials to examine the effect on participant’s age. We found that Ashwagandha extract significantly improved mental alertness on rising and anxiety but no significant effect on the QoL. In term of safety, reporting of adverse events was limited to minor side effects.

We performed a comprehensive and extensive literature review to assess the effect of Ashwagandha extract on sleep in adults. In this review, we included five trials with 400 participants, with only one trial that studied elderly adults. With the limited data, the findings of this review might not be applicable to the elderly population. Besides, trials published in the non-English language were not included. We might unintentionally miss unpublished reports or trials published in languages other than English. However, a recent meta-epidemiological study showed that excluding non-English studies from systematic reviews does not change conclusions [57].

There are some limitations to this review that are worth noting. There are many tools available to assess sleep quantity and quality, yet there is no consensus on what method should be used [58]. Three trials included in this review used actigraphy to assess sleep quality objectively. Although lab-based polysomnography is considered the gold standard for the assessment of sleep, it is not commonly used due to higher cost, more invasive, and it disrupts participants’ usual sleep routines [59]. In contrast, actigraph is cheaper, less invasive, more convenient, and has been widely used as a validated alternative to assess sleep [59–62]. While there is much evidence on the application of actigraphy, it is essential to recognize that it does not directly measure sleep. Still, it measures movement, which is then used to estimate sleep/wake cycles. Hence, the result by actigraphy may be affected by movement disorders and other conditions [63]. As for the subjective measurement of sleep quality, two tools were used in the trials. Pittsburgh Sleep Quality Index is the most widely used subjective measurement of sleep quality, and had been one of the most rigorously validated tools used in sleep diagnostics [64–66]. In this review, only two of the trials used this tool. On the other hand, the seven-point Sleep Quality Scale which was used by four of the other reviewed trials was not commonly used, and not found in previous review. Moreover, the findings from the four trials were inconsistent and the sample size was small. Hence, the findings may be an overestimation and thus required more study in the future.

The overall level of evidence in this review ranged from low to high. In general, a low or unclear risk of bias was judged for most trials in most domains. The risk of selection bias was unclear in one trial [45], as the detail of allocation concealment was not mentioned. The risk of attribution bias was present in one trial, given a relatively high drop-out rate due to non-compliance to intervention [55]. Loss to follow-up was less than 20% in the other four trials. There was no evidence of selective reporting bias. Still, we cannot exclude this because only one of the trials had published protocol and only two were prospectively registered on a trial registration database. We encountered serious inconsistency for some of our primary outcomes on sleep quality and quantity. Substantial heterogeneity was noted for overall sleep, sleep by sleep quality scale, sleep by Pittsburgh Sleep Quality Index, sleep efficiency, sleep by participant’s background with and without insomnia, sleep by treatment dosage ≥600 mg/day, and treatment duration ≥8 weeks. Serious imprecision was found on most of our primary outcomes due to the small sample size of the trials included in this review.

We attempted to reduce publication bias by checking the reference lists of all related studies for further references and searching multiple databases without restriction on publication date. However, we cannot be certain that we have located all the trials in this area. We contacted Ashwagandha extract manufacturers for further trials, but we have had no response to date. Due to limited trials, we could not construct a funnel plot for detecting publication bias.

To date, there is no other systematic review and meta-analysis that has explicitly examined the effect of Ashwagandha extract on sleep outcome in human trials. These meta-analysis results are partly in line with previous systematic reviews that examine Ashwagandha extract in other health aspects. Lopresti et al. recently published a systematic review that examined the effect of Ashwagandha extract on health and physical/cognitive performance in human trials [67]. Out of the 41 human trials that were reviewed, seven trials were on stress and anxiety, one trial on insomnia, and one trial on general well-being. Three of the trials were included in our review [53–55], and Lopresti et al. concluded that Ashwagandha extract has a positive effect on anxiety, insomnia, and general well-being. In this review, we found that Ashwagandha extract had a significant effect on sleep outcome and anxiety but no significant effect on the QoL. Besides, our result on improvement in anxiety was similar to a previous systematic review that examined the effect of Ashwagandha extract on anxiety [36].

In term of safety, reporting of adverse events was limited to minor side effects. Therefore, Ashwagandha extract seems to be relatively safe for use in improving sleep. This is in line with the finding from previous exploratory study [40].

### Authors’ conclusion

Ashwagandha extract appears to have a beneficial effect in improving sleep, both subjectively and objectively, in adults. In this meta-analysis, KSM-66 and Shoden® were the two Ashwagandha extract used. KSM-66 was used in four out of the five included trials. As it seems to be a relatively safe intervention, it can be considered an option in improving sleep until new evidence is available. Ashwagandha extract with a dosage ≥600 mg/day and treatment duration ≥8 weeks seem more effective. However, data in this review on the serious adverse effects of Ashwagandha extract are limited, and more safety data would be needed to assess whether it would be safe for long-term use.

If further research were to be done to examine the effect of Ashwagandha extract on sleep, the standardized Ashwagandha extract preparation, dosage, and duration of treatment should be determined. There are various brands of Ashwagandha extract in different form and dosage in the market, and hence standardization of extract preparation and dosage would be beneficial. The trials that we included in this review were conducted, on average, for eight weeks. Thus, there is a paucity of clinical evidence regarding the long-term safety and efficacy of Ashwagandha extract on sleep. Besides, only two trials combined subjective and objective sleep assessments in this review. Further research should use valid subjective and objective sleep measurement tools to produce a more validated outcome [59]. The lack of data on the effects of Ashwagandha extract usage in the elderly warranted that more research is needed on this population since insomnia is more common in this age group [68].

## Supporting information

S1 Checklist The PRISMA checklist. (DOC)

S1 File Search strategy. (DOCX)

S2 File Systematic review protocol. (PDF)
