# Supplementary material for: Effect of Ashwagandha (Withania somnifera) extract on sleep: A systematic review and meta-analysis
Source: PLoS One. 2021 Sep 24;16(9):e0257843. doi: 10.1371/journal.pone.0257843 (PMC8462692; doi:10.1371/journal.pone.0257843)
Supplement: S1 File — (DOCX) [file pone.0257843.s002.docx]

**Search strategy**

**Central Cochrane**

#1 ashwagandha

#2 Ashwagandha OR Withanias OR Withania somnifera OR Withania somniferas OR somniferas, Withania OR Indian ginseng

#3 #1 OR #2

#4 sleep OR difficulty sleeping OR insomnia OR insomnias OR sleep initiation and maintenance disorders OR DIMS

#5 quality of life OR life quality

#6 #4 OR #5

#7 #3 AND #6

**MEDLINE**

1. ashwagandha

2. Ashwagandha OR Withanias OR Withania somnifera OR Withania somniferas OR somniferas, Withania OR Indian ginseng

3. or/1-2

4. sleep

5. difficulty sleeping OR insomnia OR insomnias OR sleep initiation and maintenance disorders OR DIMS

6. quality of life OR life quality

7. or/4-6

8. 2 and 7
