# Supplementary material for: Effect of Ashwagandha (Withania somnifera) extract on sleep: A systematic review and meta-analysis
Source: PLoS One. 2021 Sep 24;16(9):e0257843. doi: 10.1371/journal.pone.0257843 (PMC8462692; doi:10.1371/journal.pone.0257843)
Supplement: S2 File — (PDF) [file pone.0257843.s003.pdf]

Systematic review

1. * Review title.

Give the title of the review in English

Effect of Ashwagandha (Withania somnifera) Extract on Sleep: A Systematic Review and Meta-Analysis

2. Original language title.

For reviews in languages other than English, give the title in the original language. This will be displayed with the English language title.

3. * Anticipated or actual start date.

Give the date the systematic review started or is expected to start.

04/01/2021

4. * Anticipated completion date.

Give the date by which the review is expected to be completed.

31/01/2022

5. * Stage of review at time of this submission.

Tick the boxes to show which review tasks have been started and which have been completed. Update this field each time any amendments are made to a published record.

Reviews that have started data extraction (at the time of initial submission) are not eligible for inclusion in PROSPERO. If there is later evidence that incorrect status and/or completion date has been supplied, the published PROSPERO record will be marked as retracted.

This field uses answers to initial screening questions. It cannot be edited until after registration.

The review has not yet started: No

Review stage	Started	Completed
Preliminary searches	Yes	No
Piloting of the study selection process	No	No
Formal screening of search results against eligibility criteria	No	No
Data extraction	No	No
Risk of bias (quality) assessment	No	No
Data analysis	No	No

Provide any other relevant information about the stage of the review here.

6. * Named contact.

The named contact is the guarantor for the accuracy of the information in the register record. This may be any member of the review team.

Kae Ling Cheah

Email salutation (e.g. "Dr Smith" or "Joanne") for correspondence:

Dr Cheah

7. * Named contact email.

Give the electronic email address of the named contact.

kaelingcheah@gmail.com

8. Named contact address

Give the full institutional/organisational postal address for the named contact.

Universiti Sains Malaysia, Kubang Kerian, Malaysia

9. Named contact phone number.

Give the telephone number for the named contact, including international dialling code.

60175533486

10. * Organisational affiliation of the review.

Full title of the organisational affiliations for this review and website address if available. This field may be completed as 'None' if the review is not affiliated to any organisation.

Universiti Sains Malaysia, Kubang Kerian, Kelantan

Organisation web address:

www.usm.my

11. * Review team members and their organisational affiliations.

Give the personal details and the organisational affiliations of each member of the review team. Affiliation refers to groups or organisations to which review team members belong. **NOTE: email and country now MUST be entered for each person, unless you are amending a published record.**

Dr Kae Ling Cheah. Universiti Sains Malaysia, School of Medical Sciences, Kubang Kerian, Malaysia
Dr Lili Husniati Yaacob. Universiti Sains Malaysia, School of Medical Sciences, Kubang Kerian, Malaysia
Dr Razlina Abdul Rahman. Universiti Sains Malaysia, School of Medical Sciences, Kubang Kerian, Malaysia
Assistant/Associate Professor Norhayati Mohd Noor. Universiti Sains Malaysia, School of Medical Sciences, Kubang Kerian, Malaysia

12. * Funding sources/sponsors.

Details of the individuals, organizations, groups, companies or other legal entities who have funded or

sponsored the review.

No funding/sponsors

Grant number(s)

State the funder, grant or award number and the date of award

13. * Conflicts of interest.

List actual or perceived conflicts of interest (financial or academic).

None

14. Collaborators.

Give the name and affiliation of any individuals or organisations who are working on the review but who are not listed as review team members. **NOTE: email and country must be completed for each person, unless you are amending a published record.**

15. * Review question.

State the review question(s) clearly and precisely. It may be appropriate to break very broad questions down into a series of related more specific questions. Questions may be framed or refined using PI(E)COS or similar where relevant.

What is the effect of Ashwagandha (Withania somnifera) extract on sleep in adults?

16. * Searches.

State the sources that will be searched (e.g. Medline). Give the search dates, and any restrictions (e.g. language or publication date). Do NOT enter the full search strategy (it may be provided as a link or attachment below.)

Electronic searches:

We will search the Cochrane Central Register of Controlled Trials CENTRAL (latest Issue) and MEDLINE (1966 to present).We will use the search strategy in Appendix 1 to search MEDLINE and CENTRAL. We will adapt the search strategy for other databases.

Searching other resources:

We will check the reference list of identified RCTs and review articles in order to find unpublished trials or trials not identified by electronic searches. We will also contact experts in the field and pharmaceutical companies which market influenza vaccine to identify unpublished trials. We will search for ongoing trials through the World Health Organization (WHO) International Clinical Trials Registry Platform (ICTRP) http://www.who.int/ictrp/en/ and www.ClinicalTrials.gov.

17. URL to search strategy.

Upload a file with your search strategy, or an example of a search strategy for a specific database, (including the keywords) in pdf or word format. In doing so you are consenting to the file being made publicly accessible. Or provide a URL or link to the strategy. Do NOT provide links to your search **results**.

Alternatively, upload your search strategy to CRD in pdf format. Please note that by doing so you are consenting to the file being made publicly accessible.

Do not make this file publicly available until the review is complete

18. * Condition or domain being studied.

Give a short description of the disease, condition or healthcare domain being studied in your systematic review.

Effect of Ashwagandha extract on sleep will be studied in this review.

In many modern-societies, studies have reported decline in both the sleep duration and sleep quality over the years. This is alarming as there are increasing evidence of consequences of short sleep duration. Several systematic reviews have reported the association between short sleep duration and the increased risk of hypertension (Guo et al., 2013, Wang et al., 2012), type 2 diabetes mellitus (Shan et al., 2015), obesity (Bacaro et al., 2020), metabolic syndrome, (Xi et al., 2014), coronary heart disease (Wang et al., 2016), stroke (Li et al., 2016) and a significant increase in mortality (Itani et al., 2017). Due to possible side effects from currently available pharmacological treatment for sleep disorder (Cunnington and Junge, 2016), evaluation of the effects and safety of Ashwagandha on sleep can provide a basis for decisions of use.

19. * Participants/population.

Specify the participants or populations being studied in the review. The preferred format includes details of both inclusion and exclusion criteria.

Individuals over 18 years old of any gender with or without insomnia, individuals with insomnia diagnosed by standard diagnostic criteria such as Diagnostic and Statistical Manual of Mental Disorders (DSM).

20. * Intervention(s), exposure(s).

Give full and clear descriptions or definitions of the interventions or the exposures to be reviewed. The preferred format includes details of both inclusion and exclusion criteria.

Ashwagandha extract in any form, of any dose and any duration.

21. * Comparator(s)/control.

Where relevant, give details of the alternatives against which the intervention/exposure will be compared (e.g. another intervention or a non-exposed control group). The preferred format includes details of both inclusion and exclusion criteria.

Placebo or no treatment.

22. * Types of study to be included.

Give details of the study designs (e.g. RCT) that are eligible for inclusion in the review. The preferred format includes both inclusion and exclusion criteria. If there are no restrictions on the types of study, this should be stated.

Randomised controlled trials.

23. Context.

Give summary details of the setting or other relevant characteristics, which help define the inclusion or exclusion criteria.

24. * Main outcome(s).

Give the pre-specified main (most important) outcomes of the review, including details of how the outcome is defined and measured and when these measurement are made, if these are part of the review inclusion criteria.

Primary outcomes:

1. Sleep parameters (including sleep onset latency, sleep efficiency, total sleep time, total bed time, wake after sleep onset)

* Measures of effect

Please specify the effect measure(s) for you main outcome(s) e.g. relative risks, odds ratios, risk difference, and/or 'number needed to treat.

Not applicable

25. * Additional outcome(s).

List the pre-specified additional outcomes of the review, with a similar level of detail to that required for main outcomes. Where there are no additional outcomes please state 'None' or 'Not applicable' as appropriate to the review

Secondary outcomes:

1. Mental alertness

2. Quality of life

3. Anxiety level

4. Adverse effects (such as gastrointestinal upset, impairment in renal function and impairment in liver function)

* Measures of effect

Please specify the effect measure(s) for you additional outcome(s) e.g. relative risks, odds ratios, risk difference, and/or 'number needed to treat.

Not applicable

26. * Data extraction (selection and coding).

Describe how studies will be selected for inclusion. State what data will be extracted or obtained. State how this will be done and recorded.

Using data extraction form, from each of the selected trials we will extract:

• Study setting;

• Participant characteristics (age, sex, ethnicity);

• Methodology (number of participants randomized and analyzed, duration of follow-up);

• Form, dose and duration of Ashwagandha extract used;

• Occurrence of related adverse events.

27. * Risk of bias (quality) assessment.

State which characteristics of the studies will be assessed and/or any formal risk of bias/quality assessment tools that will be used.

We will assess the risk of bias based on random sequence generation, allocation concealment, blinding of participants and personnel, blinding of outcome assessors, completeness of outcome data, selectivity of outcome reporting and other bias (Higgins 2019). We will resolve any disagreements by discussion.

28. * Strategy for data synthesis.

Describe the methods you plan to use to synthesise data. This **must not be generic text** but should be **specific to your review** and describe how the proposed approach will be applied to your data. If meta-analysis is planned, describe the models to be used, methods to explore statistical heterogeneity, and software package to be used.

Data synthesis

We plan to undertake meta-analyses using Review Manager 5.4 software (RevMan 2020) and will use random-effects model to pool data. Thresholds for the interpretation of the I^2 statistic can be misleading, since the importance of inconsistency depends on several factors. We plan to use guide to interpretation of heterogeneity as outlined: 0% to 40% might not be important; 30% to 60% may represent moderate heterogeneity; 50% to 90% may represent substantial heterogeneity; and 75% to 100% would be considerable heterogeneity (Higgins 2019).

Assessment of heterogeneity:

We will assess the presence of heterogeneity in two steps. First, we will assess obvious heterogeneity at face value by comparing populations, settings, interventions and outcomes. Second, we will assess statistical heterogeneity by means of I^2 statistic (Higgins 2019).

Measures of treatment effect:

We will measure treatment effect for dichotomous outcomes using risk ratios (RRs) and absolute risk reduction, and for continuous outcomes we will use mean differences (MDs); both with 95% confidence intervals (CIs).

Unit of analysis issues:

We will check included trials for unit of analysis errors. Unit of analysis errors can occur when trials randomize participants to intervention or control groups in clusters, but analyze the results using the total number of individual participants. We will adjust results from trials showing unit of analysis errors based on the mean cluster size and intra-cluster correlation coefficient (Higgins 2019).

Dealing with missing data:

We will contact original trial authors to request missing or inadequately reported data. We will perform analyses on available data in the event that missing data are not available.

Sensitivity analysis:

We will perform sensitivity analysis to investigate impact of risk of bias for sequence generation and allocation concealment of included studies.

Reporting biases:

If there are sufficient studies, we will use funnel plots to assess the possibility of reporting biases or small study biases, or both.

29. * Analysis of subgroups or subsets.

State any planned investigation of ‘subgroups’. Be clear and specific about which type of study or participant will be included in each group or covariate investigated. State the planned analytic approach.

If possible, we will conduct subgroup analyses on

1. Patient age (example 18-65 years and over 65 years old)

2. Patient’s characteristic (example with insomnia or without insomnia)

3. Form of Ashwagandha extract (example in pill or capsule or liquid)

4. Dose of Ashwagandha extract administration

5. Duration of Ashwagandha extract administration

30. * Type and method of review.

Select the type of review, review method and health area from the lists below.

Type of review

Cost effectiveness
No

Diagnostic
No

Epidemiologic
No

Individual patient data (IPD) meta-analysis
No

Intervention
Yes

Meta-analysis
Yes

Methodology
No

Narrative synthesis
No

Network meta-analysis
No

Pre-clinical
No

Prevention
No

Prognostic
No

Prospective meta-analysis (PMA)

No

Review of reviews
No

Service delivery
No

Synthesis of qualitative studies
No

Systematic review
Yes

Other
No

Health area of the review

Alcohol/substance misuse/abuse
No

Blood and immune system
No

Cancer
No

Cardiovascular
No

Care of the elderly
No

Child health
No

Complementary therapies
Yes

COVID-19
No

Crime and justice
No

Dental
No

Digestive system
No

Ear, nose and throat
No

Education
No

Endocrine and metabolic disorders
No

Eye disorders
No

General interest
No

Genetics
No

Health inequalities/health equity
No

Infections and infestations
No

International development
No

Mental health and behavioural conditions
No

Musculoskeletal
No

Neurological
No

Nursing
No

Obstetrics and gynaecology
No

Oral health
No

Palliative care
No

Perioperative care
No

Physiotherapy
No

Pregnancy and childbirth
No

Public health (including social determinants of health)
No

Rehabilitation
No

Respiratory disorders
No

Service delivery
No

Skin disorders
No

Social care
No

Surgery

No

Tropical Medicine
No

Urological
No

Wounds, injuries and accidents
No

Violence and abuse
No

31. Language.

Select each language individually to add it to the list below, use the bin icon to remove any added in error.
English

There is not an English language summary

32. * Country.

Select the country in which the review is being carried out. For multi-national collaborations select all the countries involved.

Malaysia

33. Other registration details.

Name any other organisation where the systematic review title or protocol is registered (e.g. Campbell, or The Joanna Briggs Institute) together with any unique identification number assigned by them. If extracted data will be stored and made available through a repository such as the Systematic Review Data Repository (SRDR), details and a link should be included here. If none, leave blank.

34. Reference and/or URL for published protocol.

If the protocol for this review is published provide details (authors, title and journal details, preferably in Vancouver format)

Add web link to the published protocol.

Or, upload your published protocol here in pdf format. Note that the upload will be publicly accessible.

No I do not make this file publicly available until the review is complete

Please note that the information required in the PROSPERO registration form must be completed in full even if access to a protocol is given.

35. Dissemination plans.

Do you intend to publish the review on completion?

Yes

Give brief details of plans for communicating review findings.?

36. Keywords.

Give words or phrases that best describe the review. Separate keywords with a semicolon or new line. Keywords help PROSPERO users find your review (keywords do not appear in the public record but are included in searches). Be as specific and precise as possible. Avoid acronyms and abbreviations unless these are in wide use.

Ashwagandha

37. Details of any existing review of the same topic by the same authors.

If you are registering an update of an existing review give details of the earlier versions and include a full bibliographic reference, if available.

38. * Current review status.

Update review status when the review is completed and when it is published.New registrations must be ongoing so this field is not editable for initial submission.
Please provide anticipated publication date

Review_Ongoing

39. Any additional information.

Provide any other information relevant to the registration of this review.

40. Details of final report/publication(s) or preprints if available.

Leave empty until publication details are available OR you have a link to a preprint (NOTE: this field is not editable for initial submission). List authors, title and journal details preferably in Vancouver format.

Give the link to the published review or preprint.
